# Segmentation of void defects in X-ray images of chip solder joints based on PCB-DeepLabV3 algorithm

**DOI:** 10.1038/s41598-024-61643-w

**Published:** 2024-05-24

**Authors:** Defeng Kong, Xinyu Hu, Ziang Gong, Daode Zhang

**Affiliations:** 1 School of Mechanical Engineering, Hubei University of Technology, Wuhan, China; 2 Present Address: School of Mechanical Engineering, Hubei University of Technology, Wuhan, China

**Keywords:** X-ray generator, Chip solder defects, Artificial intelligence, Image processing, Semantic segmentation, Automated X-ray inspection, Electrical and electronic engineering, Mechanical engineering

## Abstract

Defects within chip solder joints are usually inspected visually for defects using X-ray imaging to obtain images. The phenomenon of voids inside solder joints is one of the most likely types of defects in the soldering process, and accurate detection of voids becomes difficult due to their irregular shapes, varying sizes, and defocused edges. To address this problem, an X-ray void image segmentation algorithm based on improved PCB-DeepLabV3 is proposed. Firstly, to meet the demand for lightweight and easy deployment in industrial scenarios, mobilenetv2 is used as the feature extraction backbone network of the PCB-DeepLabV3 model; then, Attentional multi-scale two-space pyramid pooling network (AMTPNet) is designed to optimize the shallow feature edges and to improve the ability to capture detailed information; finally, image cropping and cleaning methods are designed to enhance the training dataset, and the improved PCB-DeepLabV3 is applied to the training dataset. The improved PCB-DeepLabV3 model is used to segment the void regions within the solder joints and compared with the classical semantic segmentation models such as Unet, SegNet, PSPNet, and DeeplabV3. The proposed new method enables the solder joint void inspection to get rid of the traditional way of visual inspection, realize intelligent upgrading, and effectively improve the problem of difficult segmentation of the target virtual edges, to obtain the inspection results with higher accuracy.

## Introduction

Thips are used in almost all logic electronic and electrical equipment. The soldering process perfectly combines the chip with the bare board to form functionally different integrated circuit boards^1^. The quality of the solder relates to the functional integrity of the entire board. Therefore, checking the quality of the solder is a key part of ensuring the quality and efficiency of IC production. Hidden defects inside the chip solder joints are one of the biggest hidden dangers of chip solder quality. With the development of non-destructive testing technology, the use of X-rays to irradiate the solder joints with radioactivity can effectively observe the morphology of the different structures inside the solder joints, which makes it possible to expose defects such as bridging, false soldering, and voids in the solder joints^2^. Solder joint cavity phenomenon is one of the most common hidden defects in solder hidden defects, If the cavity area is too large, or the number of cavities will seriously affect the quality of the solder, so it is very important to accurately detect the cavity.

The use of X-ray technology to inspect welded joints for hidden defects has gained general acceptance, and some researchers have made outstanding contributions to the advancement of this field. For example, Gao et al.^3^, through the pre-processing of X-ray images such as Gaussian filtering, contrast stretching, etc., the use of the Canny operator for edge detection to locate the edge region of the weld joint cavity, and finally the use of digital image processing algorithms to extract the edges of the bubble region inside the weld ball, to achieve high-quality detection results, although by taking some pre-processing methods for X-ray images and at the same time, applying the classical Although some pre-processing methods are adopted for the X-ray image and the classical Canny operator is also used to segment the hard-to-segment cavity edges, the fuzzy zones of the cavity edges are of different widths and shapes, which are difficult to be segmented accurately by the traditional image processing methods. Xiao et al.^4^ proposed a method that firstly adopts the OTSU adaptive segmentation algorithm to extract the weld ball region, and then uses the mathematical morphology of the open and closed operations and the top hat transform to extract the bubble region of the weld ball. The algorithm calculates the interclass variance between foreground and background based on the grayscale characteristics of the whole BAG ray image and derives a suitable threshold value that maximizes the interclass variance value to segment the welding ball bubble region. Although this method can segment the weld ball bubble region, the segmentation of fuzzy pixels at the edge of the cavity is incomplete, the problem of accurate segmentation is still not solved, and the process is cumbersome and not easy to operate. With the development of deep learning in the field of machine vision, some scholars have also begun to study the detection of X-ray weld ball void defects based on deep learning, for example, Zhang, QR et al.^5^. solved the problem of ROI noise and image size change by introducing deep learning methods to improve the adaptability of the model to different sizes of voids and the accuracy of the detection, but the method solves the problem of the existence of voids and does not measure the size of the voids. Li, LL et al.^6^. proposed an adaptive pseudo-color enhancement algorithm for high-level grayscale (super 8-bit) images to enhance high-level grayscale images and improve the clarity of defect image details, to realize the accurate localization of weld and porosity defects in solid engine casing X-ray film, and the work of this thesis enhances the contrast between defects and the background, and although it can realize the identification and localization of weld and porosity defects, it does not solve the problem of porosity defects. Recognition and localization, but did not solve the problem of segmentation of air hole defects; Xiao et al.^7^. proposed an adaptive hybrid framework for multi-scale void detection of welded joints of chip resistors, but the hybrid framework did not consider the fusion of shallow detail features and deep high semantic features, and the generalization performance of small-size voids is poor, and the network did not consider the discrete role of redundant information, and the network lacked the reinforcement of important features role, which affects the operational efficiency and robustness of the network.

The quality of chip welding brings hidden danger to the safe and stable operation of equipment terminals, and the void is the type of defect that accounts for the largest proportion of the chip welding process^8^, and the fast and accurate measurement of the void area is the basic to improve the quality of chip assembly, and the highly accurate segmentation of the chip weld node void defects based on the X-ray image is the key to realize the measurement of the void area. Traditional chip solder joint defect detection makes it difficult to achieve the measurement of the size of the cavity, and the process is cumbersome, the detection accuracy is low, and the chip solder joint cavity segmentation based on the deep learning method does not take into account the extraction of detailed edge features and the filtering of redundant information, resulting in the model Yong total, the operation efficiency is low, can not meet the engineering applications, and the cavity fuzzy edge segmentation is inaccurate and so on. To address the above problems, the PCB-DeepLabV3 image segmentation algorithm is proposed to improve the segmentation of X-ray images of solder joint cavities. The Mobilenetv2^9^ network is used as the backbone feature extraction network of DeepLabV3^10^, which ensures accuracy while obtaining a lighter network model, which is helpful for the deployment in industrial scenarios and improves the inference speed; the attentional multi-scale two-space pyramid pooling network is designed to effectively retain the detail feature information, and more detail information is helpful in the extraction of weak features on the false edges and accurate segmentation; meanwhile, during the convolution process, the detail information is lost, which leads to the loss of tiny targets and the loss of the detail information in the segmentation. At the same time, during the convolution process, the lost detail information leads to the loss of tiny targets, The attentional multi-scale two-space pyramid pooling network compensates for this defect, and more details can be captured and successfully segmented; in addition, to training a more robust deep learning model, data enhancement is carried out on the original dataset, and ultimately a better robust model is obtained, which effectively solves the problem of the scarcity of data sources in the current situation where the training data is increasingly valuable.

In this paper, white high-power LED chips packaged by surface mount technology are collected as research objects for design experiments^11^. X-ray images of the solder joints were acquired for all LED chips using the same setup on a 2D X-ray device. X-rays are mainly used to detect gas inclusions in the chip solder joints and unwetted soldered areas (un-soldered areas due to residues or contamination on the component or substrate)^12^. Both appear brighter in the X-ray image due to the lack of metal and more radiation can pass through the area to form a white image, resulting in so-called voids. The voids are first labeled into JSON data format using the Labelme data annotation tool; then the JSON training data is converted into VOC format; and finally, the segmentation of the different targets is completed by training the converted data with the improved PCB-DeepLabV3. Nowadays advanced AXI equipment has been commonly used in the integrated circuit industry^13^, with the help of X-ray penetration properties of different substances to successfully image the hidden defects, which is convenient for technicians to check the quality of solder joints. The method proposed in this paper can provide an intelligent modification to the existing automatic X-ray inspection^14^ equipment. Because AXI equipment still needs technicians to confirm visually, this paper combines the deep learning-based PCB-DeepLabV3 image segmentation algorithm to propose an automated inspection method, which gets rid of the dependence on the technician's experience and greatly improves the inspection efficiency and accuracy in the industrial scenario.

The rest of the paper is arranged as follows. Section "Introduction to the attentional multi-scale two-space pyramid pooling network (AMTPNet)" introduces the attentional multi-scale two-space pyramid pooling network (AMTPNet). Section "PCB-DeepLabV3: an improved Deeplabv3 network based on AMTPNet" introduces the improved PCB-DeepLabV3 image segmentation model. Section "PCB-DeepLabV3: an improved Deeplabv3 network based on AMTPNet" describes the dataset production and experimental setup. Section "Experimental results and discussion" trains the improved PCB-DeepLabV3 image segmentation model using the well-produced LED chip X-ray image dataset, evaluates the performance of the PCB-DeepLabV3 model and verifies the new model's ability to realistically segment X-ray images of solder joint voids. Section "Conclusion" concludes.

## Introduction to the attentional multi-scale two-space pyramid pooling network (AMTPNet)

The attentional multi-scale two-space pyramid pooling network structure proposed in this section involves two excellent network structure models, the attention network^15,16^ and the spatial pyramid pooling network^17^. Firstly, these two network structures are briefly introduced; then details are presented to draw on the two network ideas to form a new model, i.e. the attentional multi-scale two-space pyramid pooling network.

### Channel attention mechanism

In this paper, attention ideas are introduced into the multi-scale spatial dual pyramid pooling network, although the attention module can be plug-and-play, simply interspersed in the network nodes is not obvious. However, the attention idea is very important and has been proven in numerous application scenarios. The representative model of the channel attention mechanism is the compression and excitation network. The SENet^16^ is divided into two parts, compression and excitation, The purpose of the compression part is to compress the global spatial information, and then feature learning in the channel dimension to form the importance of the individual channels, and finally re-weight the individual channels by the excitation part.

### Attentional multi-scale two-space pyramid pooling network (AMTPNet) design

**Figure 1:**
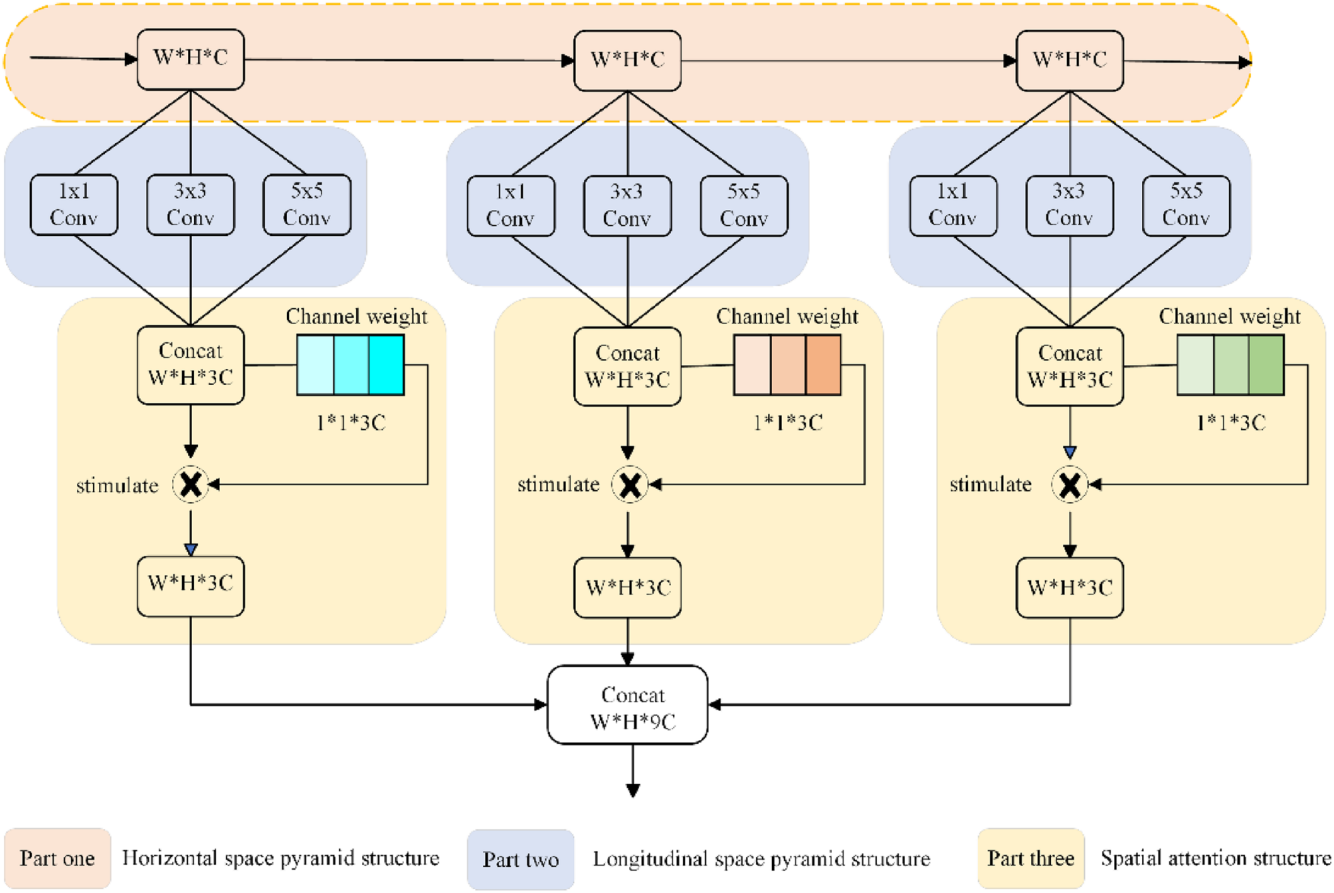
Attention multi-scale two-space pyramid network pooling structure.

Spatial pyramid pooling network (SPPNet) solves the problem that the input data must be of fixed dimensions, and at the same time fuses the different scales of pooled feature layers to improve the ability of the model to extract features. The pooling process of SPPNet leads to the loss of some spatial detail information, and to reduce this loss, the null convolution^18^ has been devised to replace the pooling operation. Null convolution can increase the sensory field, but null convolution needs to face two problems, firstly the grid effect loses the continuity of the information and secondly, the long-term information may be irrelevant. To circumvent the defects of pooling operation and null convolution, this paper adopts continuous convolution operation, to ensure that the fusion part of the dimensionality of the consistency of the stackable, the convolution process using padding. Section “Channel attention mechanism” briefly introduces the channel attention mechanism. This section combines the idea of attention and spatial pyramid pooling network to construct the attentional multi-scale two-space pyramid pooling network, and the structure of its network is shown in Fig. 1.

In Fig. 1, the attentional multi-scale two-space pyramid pooling network consists of four parts. The first part of the horizontal side space pyramid structure, it should be noted that the input layer is three different shallow feature layers, in order to ensure the subsequent spatial superposition operation, the three different feature layers need to maintain the dimensionality consistency, the main purpose of the horizontal side space pyramid structure is to obtain the rich details of the information; the second part of the longitudinal side space pyramid structure, respectively, with 1 × 1, 3 × 3, 5 × 5 size convolution kernel on the the same feature layer, in order to ensure that the size of the convolution is unchanged after the convolution, the convolution process were used padding equal to 0, 1, 2 to obtain the same feature dimensions for the next superposition fusion; the third part of the channel attention mechanism, the introduction of the channel attention mechanism to extract the feature weights, for the fusion of the feature layer in the channel dimensions of the weight value of each layer is calculated to the weight vector. Then the weight vector is used to motivate the fused feature layer, and the feature layer parameters are updated to obtain a fused feature layer with more prominent features; the fourth part adjusts the number of channels, and the number of channels is adjusted with a 1 × 1 convolution to ensure the same dimension as the initial feature layer.

## PCB-DeepLabV3: an improved Deeplabv3 network based on AMTPNet

This section focuses on the improvement of the Deeplabv3 segmentation network. The Attentional Multi-scale Two-space Pyramid pooling Network (AMTPNet) has been described in detail in Section "Introduction to the attentional multi-scale two-space pyramid pooling network (AMTPNet)", and this section focuses on how to embed the AMTPNet network into the Deeplabv3 network and integrate the MobilenetV2 model into the backbone of the Deeplabv3 network, replacing the original Xception^19^ structure to form the complete segmentation network model The MobilenetV2^9^ model is integrated into the Deeplabv3 network backbone. Firstly, the MobilenetV2 model is briefly introduced; then the Deeplabv3 network is introduced; finally, the improved PCB-Deeplabv3 model is introduced in detail.

### Mobilenetv2 model

Neural networks are getting bigger and bigger, the structure is getting more and more complex, and more hardware resources are needed for prediction and training, and deep learning neural network models can only be run in high-computing power servers. Mobile devices make it difficult to run complex deep-learning network models due to the limitations of hardware resources and computing power. From 2016 until now, lightweight network models such as SqueezeNet^20^, ShuffleNet^21^, NasNet^22^, MnasNet^23^, and MobileNet^24^ have been proposed in the industry. These models make it possible to run neural network models on mobile terminals and embedded devices. MobileNet is the most representative of lightweight neural networks, and there are three versions of the Mobilenet network, In this paper, we use Mobilenetv2 as the backbone network of Deeplabv3 semantic segmentation model to extract the main features, and the linear bottleneck structure and the inverse residual structure are introduced into MobileNetV2. MobileNetV2 has six bottlenecks in the network model. There are six Bottleneck layers in the network model, and each Bottleneck contains two point-by-point convolutional layers and one deep convolutional layer, with a total of 54 trainable parameter layers.

### Deeplabv3 network and its improvement

The Deeplabv3 network^25^ as a whole is divided into Encoder and Decoder parts. The encoder part mainly includes the backbone and ASPP^26^ parts. In the Decoder part, the low-level features from the backbone middle layer and the high-level features output from the ASPP module are received as inputs.

**Figure 2:**
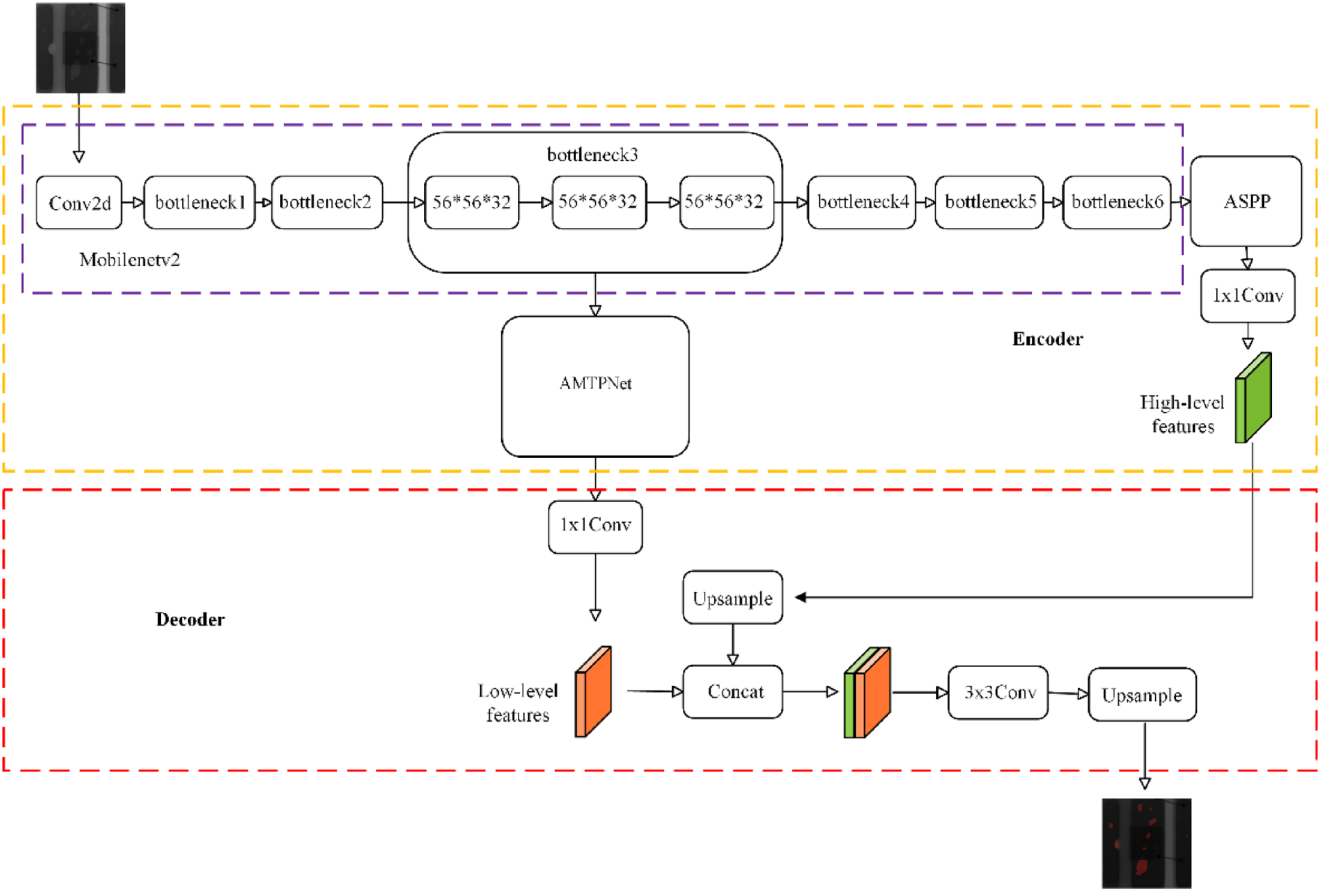
PCB-DeepLabV3 network structure.

The modified Deeplabv3 network still maintains the Encoder-Decoder structure, with the difference that it is replaced in the backbone part by the modified Mobilenetv2 network, which intercepts the first 6 Bottleneck layers and its last feature layer outputs the dimensions of 32 × 32 × 320; the most important change is in the Encoder part to add AMTPNet network for spatial double pyramid feature fusion on shallow information. The improved Deeplabv3 model is named PCB-DeepLabV3 and the network structure is shown in Fig. 2.

Observing Fig. 2, it can be seen that in the Encoder part, firstly, the 3rd bottleneck layer is taken in the backbone part, and since the 3rd bottleneck layer is repeated three times, the horizontal pyramid network structure is formed from these three low-level feature layers; then, the vertical attention space pyramid feature fusion is performed using the AMTPNet network to obtain 56 × 56 × 288 feature layer; finally, after MobileNetv2 backbone feature extraction, the ASPP network is used to enhance the feature extraction after the last convolutional feature layer in bottleneck6. In the Decoder part, firstly, the channel downsampling is performed on the feature maps output from the AMTPNet network using 1 × 1 convolution, from 288 to 48 dimensions (the reason why we need to downsample to 48). to 48 is because too many channels will mask the importance of the feature maps output from AMTPNet); then, the feature maps from the ASPP output are interpolated and upsampled to get the feature maps with the same dimensions as the low-level features; then, the low-level features and high-level features are linearly interpolated and upsampled to obtain feature maps that are stitched together using concat and fed into a set of 3 × 3 convolutional blocks for processing; finally, linearly interpolated and upsampled again to obtain predicted maps with the same resolution size as the original maps. The backbone part of the original Deeplabv3 network adopts the Xception main structure. Section “Mobilenetv2 model” has introduced the Mobilenetv2 network, to make Deeplabv3 also has the advantages of smaller size, faster speed without decreasing the accuracy, and easier deployment, etc., the Deeplabv3 network is replaced with the Mobilenetv3 network. The backbone part is replaced with the Mobilenetv2 network to achieve lightweight transformation. In the Decoder part, the three low-level features of the backbone3 layer are fused with AMTPNet, and the feature maps after attentional multiscale spatial bipyramid feature fusion are then stacked and fused with the high-semantic feature maps output from the ASPP module. This has the advantage of retaining more shallow detail information and optimizing the distribution of spatial features with better segmentation capability for edge pixels.

## PCB-DeepLabV3: an improved Deeplabv3 network based on AMTPNet

### Data set production

**Figure 3:**
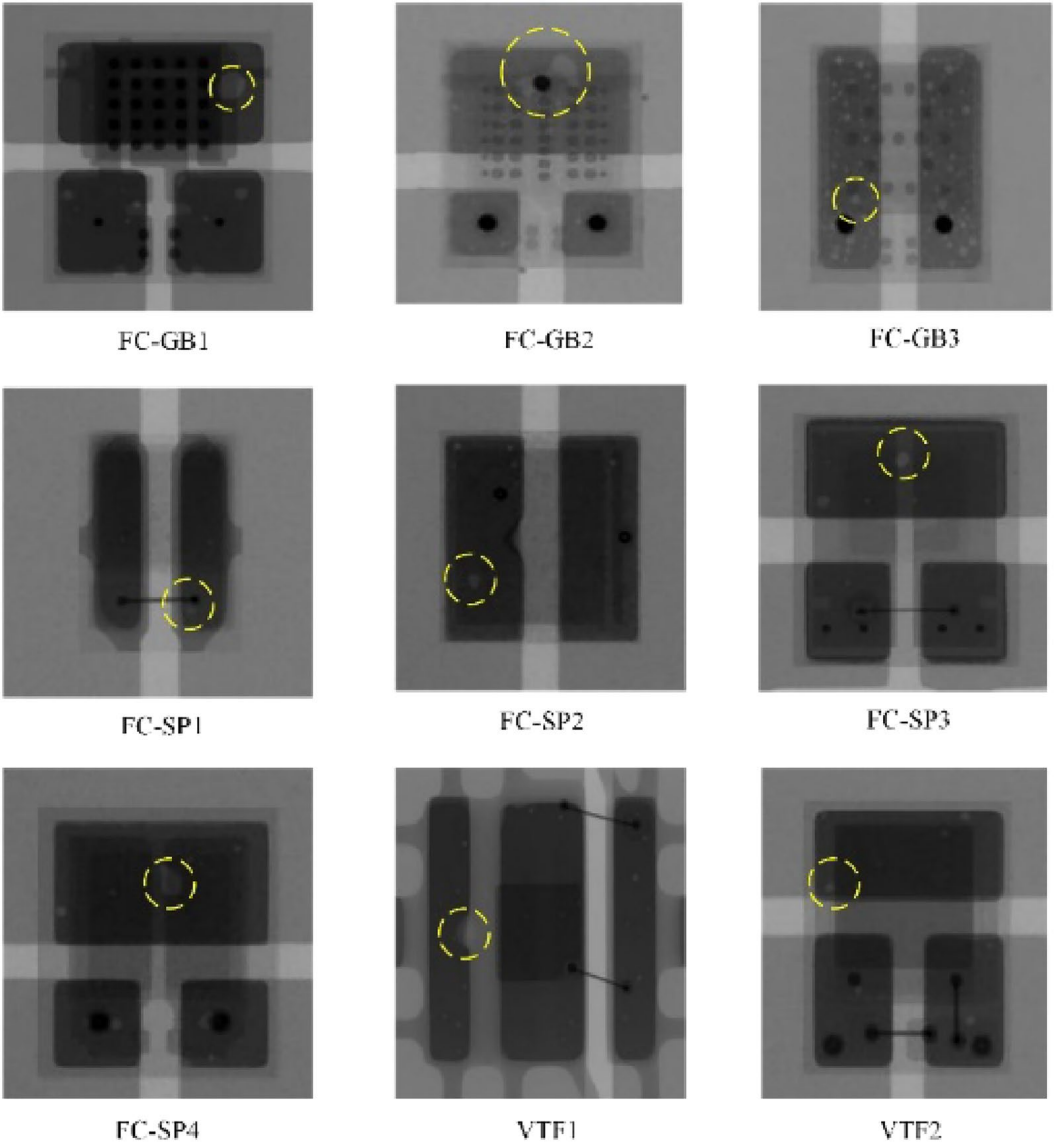
X-ray images of 9 kinds of LED chips and some hollow defects are shown.

The study objects used in this work are nine types of LED chips from seven manufacturers^14^, all packaged using surface mount technology (SMT). The nine chip fabrication materials and properties are very similar, but the mechanical dimensions, package types, and thermal properties vary greatly, thus constituting a wide range of morphology and void types. The dataset contains a total of 450 LED chips on 20 substrates, with each LED chip mounted on a designated position on the substrate with solder paste brushed on it, and all the LED chips were acquired on a 2D X-ray device using the same settings. A sample of the acquired images is shown in Fig. 3.

Figure 3, nine kinds of LED chip welding areas that can be complete and clear imaging, different structure material imaging different gray-scale images, the darker color area that is the pad, the hollow and chip beam build imaging for gray and white, which circle marked is part of the hollow area. The different internal structures of the LED chip can be generally divided into three types flip-chip with pads FC-SP class, with gold bumps flip chip FC-GB class, and vertical film VTF class. Because the internal structure is not the same, the three types of LED chip cavity layout and morphology are also different, for example, FC-SP class chip anode and cathode contacts using two flat pads connected to the base, X-ray image chip base color is darker and clearer, the cavity area is smaller and fewer in number, and the background difference with the base surface; FC-GB class chip anode and cathode of the back side of the connection is made through the base on the gold bumps rather than two soldering blocks. FC-GB class chip anode and cathode backside connection are realized through the gold bump on the base instead of two solder interconnections, to improve the mechanical stability of the chip and the base between the application of the bottom of the filler, so it will be more likely to produce voids, this type of chip void layout is diffuse, the number of sporadic and irregular; VTF class chip anode connection from the top of the layer through the lead bonding to the base of the layer contact area, the structure does not need to redistribute the layer, so there is less chance of generating voids. Most of the bubbles are laid out with the edge of the chip base surface and the number is small. Irregular voids of varying numbers and sizes are distributed on the pads, and the next task will be to label the voids. The Labelme platform is used to manually annotate into JSON file format, and then the JSON format is converted into VOC format to facilitate the training of the image semantic segmentation model.

### Dataset expansion and cleaning

**Figure 4:**
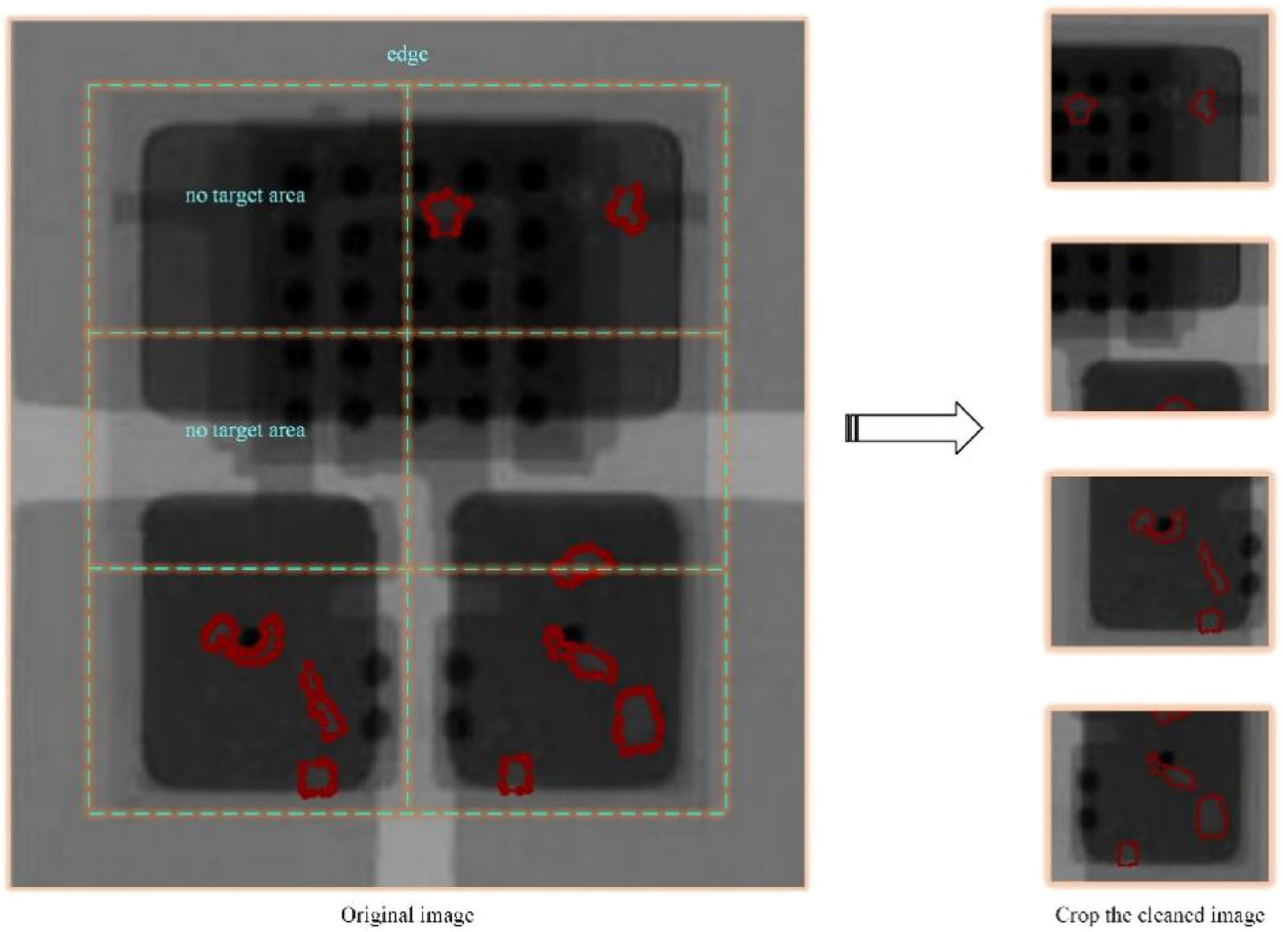
Cropping cleaning process of the original image.

Since the overall dataset has only 450 images, which is objectively a small-scale dataset, increasing the sample size of the training set is a feasible method to train a better model. In this paper, we design an image cropping method to increase the training dataset while balancing the ratio of background and foreground training samples. The comparison image before and after image cropping is shown in Fig. 4.

Observing Fig. 4, it can be seen that, firstly, the edge-side non-chip-based X-ray images in the big picture are cropped out; then, the remaining parts are equally divided into 6 parts, and if there is no bubble pixel in the cropped image, it is discarded; finally, the ratio of bubble pixels to the cropped image is calculated, and the images with a ratio of less than 5% are discarded. After the above operations, 4128 trainable images are finally obtained.

### Experimental setup and model training

This paper mentions that the training and evaluation of all the models were carried out on the open-source toolkit PyTorch via the Pycharm platform on a computer with a 12th Gen Intel^®^ Core™ i5-12600KF 3.70 GHz CPU and 16 GB of installed RAM. They are using an NVIDIA GeForce RTX on Windows 10 3070 GPU (with 8 GB of RAM) to run all the semantic segmentation models involved in the experiments. After non-stop exploratory experiments to summarise the important hyperparameters in the semantic segmentation model, the model achieves stable and reliable performance when the model parameters are set as in Table 1.

**Table 1:** Experimental setup.

Set item	Parameter
Iteration	100
Batch size	4
Initial learning rate	0.007
Min learning rate	0.0001
Optimizer	SGD
momentum	0.9
Weight decay	0.0001
Learning rate decay type	COS
Thread	4

During model training, the number of images processed at one time (Batch Size) is set to 4, the optimizer selects SGD and sets the initial learning rate to 0.007, the learning rate decreases by cosine "COS", and the weight decay rate is set to 0.0001, to avoid model overfitting. If the validation loss does not decrease for 3 epochs, the training is stopped. After many experiments, when the model is iterated for 100 generations, the loss value tends to converge successfully, so the number of training iterations is set to 100 to meet the requirements.

## Experimental results and discussion

**Figure 5:**
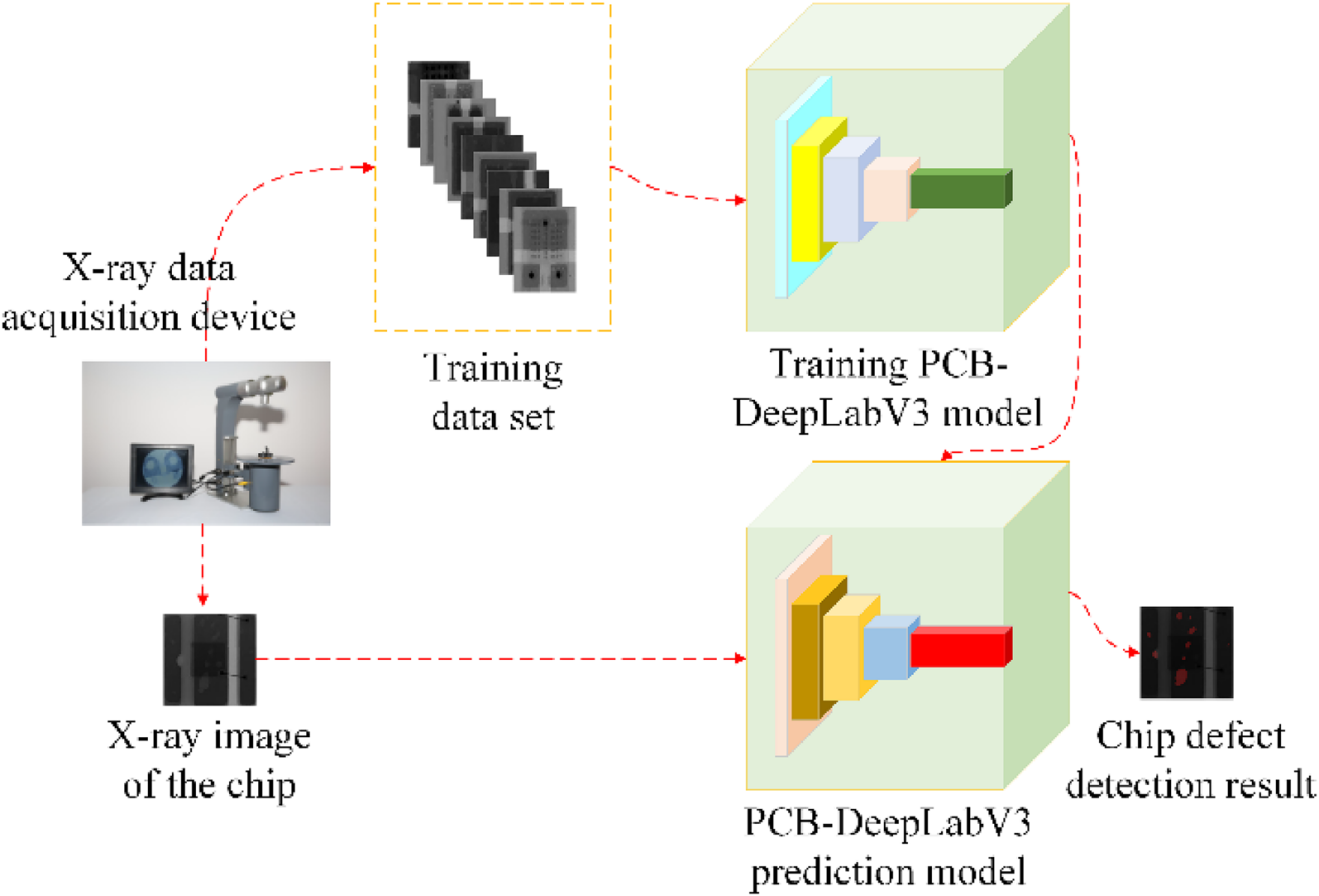
Chip void defect inspection complete process.

The experimental model and manufacturing engineering deployment schematic proposed in this paper are shown in Fig. 5.

Three phases of data acquisition, model training, and engineering deployment of the mature model are included in Fig. 5. In the data acquisition phase, it is done by an industrial X-ray collector; in the training phase, the PCB-DeepLabV3 model is trained by feeding the model with artificially labeled chip X-ray images until the model converges; in the engineering deployment phase, the successfully trained model is deployed in a chip manufacturing industrial scenario to complete the automated quality inspection.

**Figure 6:**
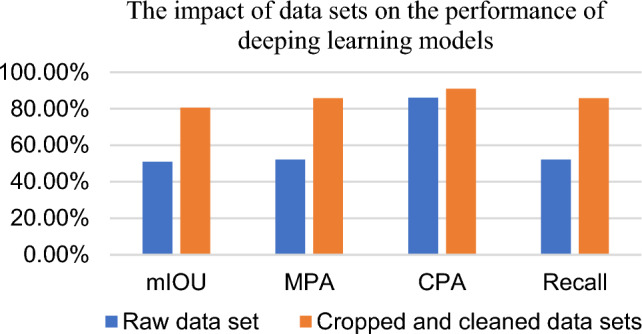
Impact of dataset on deep learning model performance.

To verify the importance of the dataset to the deep learning model, the original data and the cropped and cleaned data are input into the DeeplabV3 model respectively and are trained under the same experimental platform and settings respectively, after the training is completed, the two mature models are obtained and evaluated for their performance. Semantic segmentation is pixel-level classification, and its common evaluation metrics are three metrics such as mIOU (mean intersection over union), MPA (mean pixel accuracy), and CPA (Class Pixel Accuracy), where MPA and CPA correspond to the average accuracy in the classification model (mAP) and Precision (Precision) in the classification model, Recall indicator is not a commonly used indicator in semantic segmentation model, but in this paper, to illustrate the completeness of the empty pixel segmentation, so the Recall indicator is chosen, and the test results are shown in Fig. 6.

The bar chart in Fig. 6 shows that the model trained on the cropped and cleaned dataset substantially outperforms the model trained on the original dataset in the four metrics of mIOU, MPA, CPA, and Recall, which shows the importance of cropping and cleaning the original dataset for deep learning models. The principle of this is explained as follows: firstly, the sum of pixels of the six small images after cropping is smaller than that of the complete large image, and the non-chip-based X-ray image on the edge side of the large image is discarded, which is not useful for training the segmentation of foreground region and exacerbates the proportion of foreground and background samples in the training; however, the serious imbalance in the proportion of the samples makes it easier for the model to ignore the classes with a lower proportion and thus make the model invalid; then, the original dataset is cropped and cleaned to make the model more effective. Then, images with no foreground pixels after cropping and images with a lower proportion of foreground pixels are discarded to further reduce the foreground-to-background sample ratio, and a more balanced class-to-sample ratio helps the model learn the features in the foreground, which is important for the model to learn the semantics of segmenting the pixels in the empty region.

**Figure 7:**
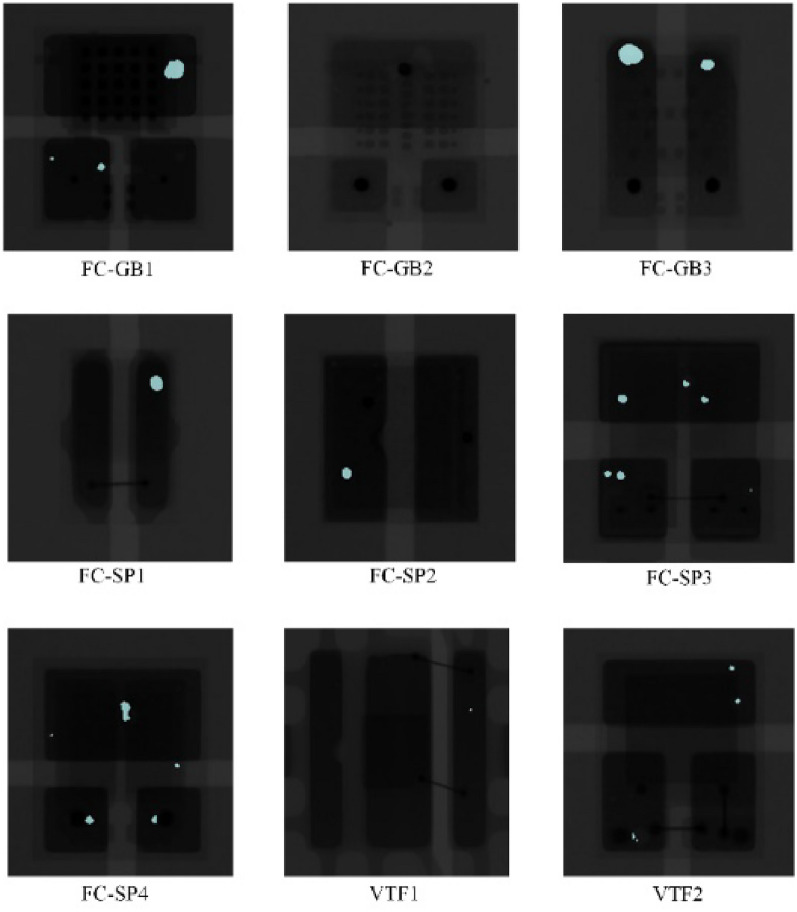
X-ray images of 9 kinds of LED chips in DeeplabV3 model test result display.

The cleaned LED chip X-ray dataset is input into the DeeplabV3 model and trained according to the experimental platform prepared in Sect. “Dataset expansion and cleaning”. After the training is completed, the nine LED chip X-ray images are tested, and the test results are shown in Fig. 7.

**Figure 8:**
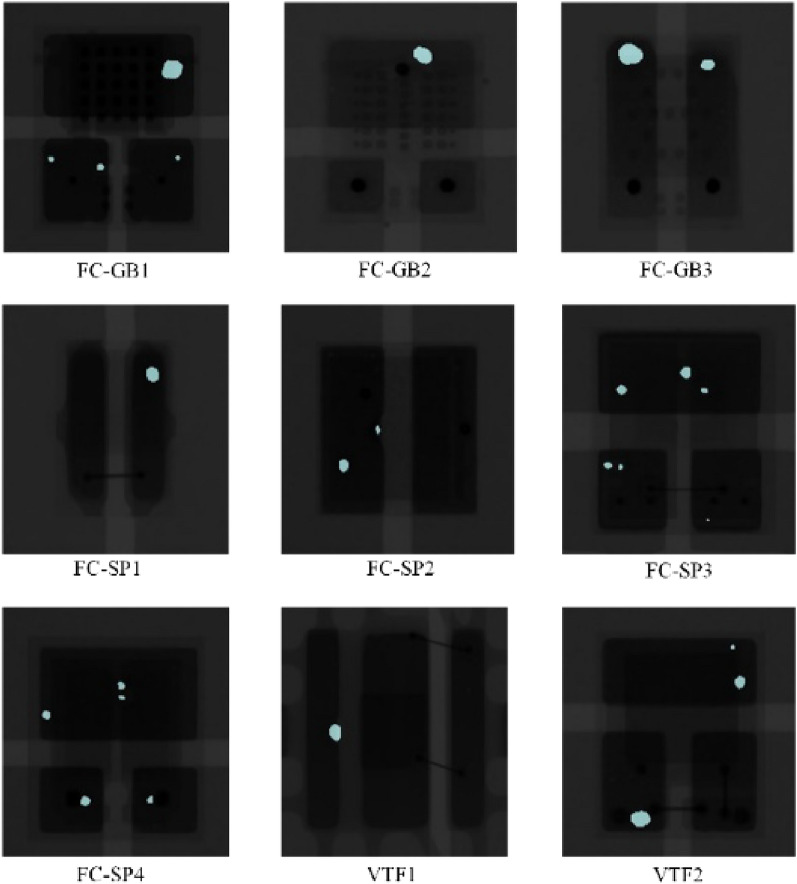
X-ray images of 9 kinds of LED chips in PCB-DeeplabV3 model test result display.

Observing Fig. 7, it can be seen that the DeeplabV3 model has a certain segmentation ability for 9 kinds of LED chip void defects. Due to the formation of fuzzy virtual edges between the voids and the soldered surface, and the low contrast between the FC-GB2 model chip voids and the base surface of the soldered joints, the above factors make it difficult to accurately segment the void defects of different LED chips. Through the actual testing of the DeeplabV3 model, it is found that most types of cavity segmentation are incomplete, especially in the difficult to segment FC-GB2 model chip cavity segmentation effect is not ideal, and model failure problems. By introducing the improved PCB-DeeplabV3 model, the model is trained in the same experimental environment and the same samples are used to test the model performance. The test results are shown in Fig. 8.

**Figure 9:**
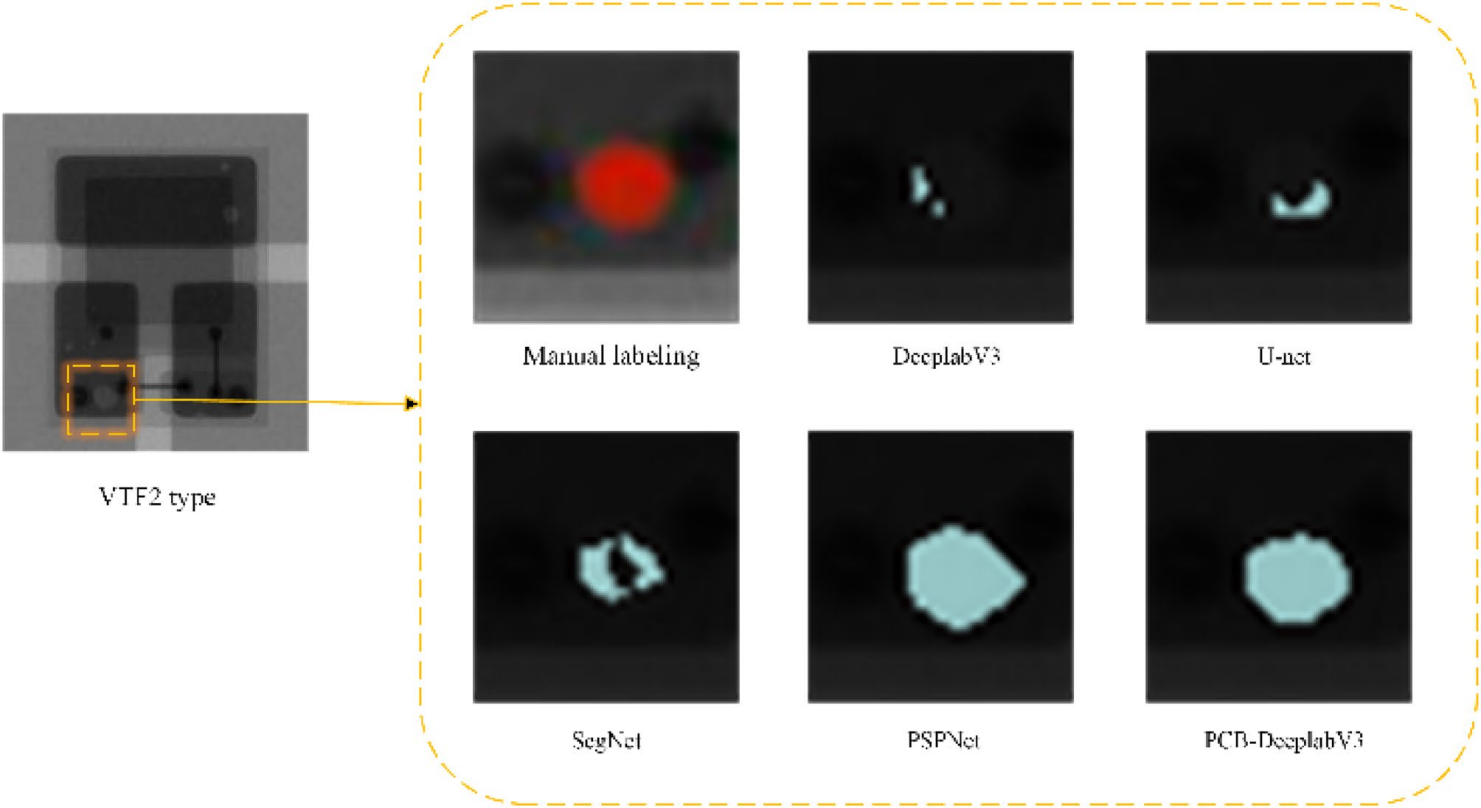
Comparison of local details of typical bubble defect segmentation results by different methods.

Figure 8 the only variable of the experiment is the semantic segmentation model, and observation of Fig. 8 reveals that the segmentation performance of the improved PCB-DeeplabV3 model is significantly improved, and the segmented void area region is larger than that segmented by the DeeplabV3 model for most of the chip types, and the void with smaller area is also detected, especially in the case of FC-GB2 model chip with low contrast, which can still be successfully segmented, indicating that the proposed AMTPNet network helps the DeeplabV3 model to improve the segmentation performance for hard-to-segment voxels and low-contrast pixels. The next experiment compares the performance of the improved PCB-DeeplabV3 model with DeeplabV3, U-net^27^, SegNet^28^, and PSPNet^29^, so that it is easier to choose the appropriate model for deployment in real engineering applications. In this part of the experiment, the VTF2 model chip is selected as the basic experimental object, and concerning the manually labeled images, the comparison of the segmentation performance of different models in the most critical void defect detail part is shown in Fig. 9.

In Fig. 9, when the detection model is VTF2, the area segmented by Deeplabv3, U-net, SegNet, PSPNet and the method in this paper is compared with the manually labelled area, and the changes in the detail part are observed to see the better detection capability of the PCB-DeeplabV3 detection results compared to the above models, which is mainly reflected in the increase in the segmented area and area Coherence, for example, Deeplabv3, U-net model lost a lot of empty defect areas, SegNet model appeared serious incoherence phenomenon, PSPNet model, although the detection of the bubble area greatly increased, but has exceeded the actual bubble defect area, segmentation of excessive, the method in this paper can basically accurately detect the bubble defects, and difficult to segmentation of the virtual edge of the spatial structure of a better spatial deconstruction ability, which also indicates that the model in this paper can handle better in ambiguous edge details and has better performance in resolving edge pixels.

To verify the performance impact of the channel attention mechanism (CAM), the two-space pyramid pooling network (TSP), and their fused network structure (AMTPNet) on the semantic segmentation model, the following ablation experiments provide a point-by-point comparison of the improved parts by the control variable method. This experiment uses the dataset enhanced by the cropping and cleaning method to train the model, using Deeplabv3 as the baseline model. Model a is the channel attention mechanism alone; model b is the two-space pyramid pooling network alone; and model c is the fusion network structure that considers both, and the results of the ablation experiments are shown in Table 2.

**Table 2:** Effect of different modules on model performance.

Model	Module		Evaluation index			
	CAM	TSP	mPA (%)	mIOU (%)	CPA (%)	Recall
Standard	✕	✕	85.73	79.26	86.27	82.73
a (CAM_Deeplabv3)	√	✕	85.80	79.75	88.29	83.83
b (TSP_Deeplabv3)	✕	√	85.82	80.21	88.36	84.99
c (AMTPNet_Deeplabv3)	√	√	85.88	81.69	90.09	85.96

In this experiment, the void is used as a positive sample, and all the background except the void is used as a negative sample, separating the positive sample void from the negative sample background pixels, which is essentially a binary classification problem. Observing the mPA, mIOU, CPA, and Recall metrics data for models a, b, and c in Table 2, the addition of the CAM and TSP modules to the baseline model DeeplabV3 improves all metric values to a certain extent, indicates that the individual modules all play an active role in the model; when using the AMTPNet structure, the mIOU and mPA are once again improved, It shows that the channel attention mechanism (CAM) and the two-space pyramid pooling network (TSP) have good combinability and promote each other for better performance. For binary classification problems, especially when the model focuses on the party that is in the minority in the binary classification, the accuracy (mPA) loses its significance in judging, and the more commonly used evaluation metrics are the precision (CPA) and the recall (Recall). The mIOU, CPA, and Recall evaluation metrics in Table 2 are highly variable, and the CPA metrics improve more substantially when the baseline model is used with different models a, b, and c. This suggests that the Channel Attention Mechanism (CAM) and the Twin Spatial Pyramid Pooling Network (TSP) practically improve the accuracy of the model in segmenting pixels representing voids and that the combination of the two, the AMTPNet structure fusion performance is better, and the improvement of Recall metrics also proves the positive effect of models a, b, and c on the overall network performance improvement.

To verify that this paper's model also has advantages in the post-deployment inference process, this paper's method is compared with other major classical models, and two indexes, mIOU and FPS, are used as evaluation criteria. Where mIOU refers to the average of the intersection and merge ratio of each class in the dataset, and the intersection and merge ratio is the ratio of the intersection and merge of the true label and the predicted value of the class, the larger the ratio, the more accurate the segmentation is; FPS refers to the number of frames transmitted per second, which can measure the model inference speed, and the larger the value is, the higher the speed of the model inference is. Table 3 records the mIOU and FPS values for U-net, SegNet, PSPNet, DeeplabV3, and the method in this paper under the same experimental conditions.

**Table 3:** Values of mIOU and FPS for different models.

Model	mIOU (%)	FPS
U-net	68.19	70.65
SegNet	74.36	64.26
PSPNet	79.58	74.34
DeeplabV3	62.47	68.42
Ours	81.69	78.81

As can be seen from Table 3, the method proposed in this study outperforms other methods in both mIOU and FPS metrics. Among them, the average intersection and merger ratio reaches 81.69%, and the model inference speed reaches 78.81 FPS, which indicates that the AMTPNet network improves the accuracy of model segmentation, especially on the fuzzy edge structure that is more difficult to segment, and at the same time, the backbone feature extraction part adopts the lightweight MobileNetV2 network, which accelerates the model inference speed and improves the performance of the model for practical deployment applications.

## Conclusion

In this study, based on the Deeplabv3 network model, MobileNetV2 is introduced to optimize the network parameters so that the model is reduced in size and easy to deploy, and based on this, attentional multi-scale two-space pyramid pooling network (AMTPNet) is embedded into the low-level features, which facilitates the fusion of the edge details and extraction of the model. In the process of model training, the data set is enlarged and the proportion of positive and negative samples is reduced by cropping and cleaning the original data so that the model can be adequately trained in a small data set while eliminating the negative impact of the imbalance of the proportion of positive and negative samples. Through the above series of experiments and analyses, and comparing the improved PCB-Deeplabv3 with the current excellent U-net, SegNet, PSPNet, and DeeplabV3 classical semantic segmentation models, the following conclusions can be drawn:

- When training the deep learning model, the image cropping and cleaning method designed in this paper greatly enhances the training dataset so that the model is fully trained to fit the target successfully, and there is a substantial improvement in all 4 metrics of mIOU, MPA, CPA, and Recall.
- The AMTPNet network proposed in this study has a better effect on the extraction of detailed information that is difficult to segment at the edge, has a better generalization ability in identifying X-ray images of different chip models, and successfully detects void defects.
- The PCB- Deeplabv3 model proposed in this paper has better segmentation performance compared with Unet, SegNet, PSPNet, DeeplabV3 model, especially in the blurred edges and low-contrast LED chip X-ray void defect images, the PCB- Deeplabv3 model proposed in this paper has better performance, and reaches 81.69% average accuracy in the mIOU and FPS indexes reaching 81.69% average accuracy and 78.81 frame rate, respectively, surpassing the performance of other classical models.

## Data Availability

The datasets generated during and/or analyzed during the current study are available in the [X-ray-Void-Defect-Detection-in-Chip-Solder-Joints-Based-on-PCB-DeepLabV3-Algorithm] repository, [https://github.com/jackong180/X-ray-Void-Defect-Detection-in-Chip-Solder-Joints-Based-on-PCB-DeepLabV3-Algorithm]. The datasets generated and/or analyzed during the current study are available in the [Reliability of High-Power LEDs and Solder Pastes] repository, [https://www.kaggle.com/datasets/andreaszippelius/hellastudy-of-leds2].
